# Effect of evidence-based drug therapy on long-term outcomes in patients discharged after myocardial infarction: a nested case–control study in Italy†‡§

**DOI:** 10.1002/pds.3430

**Published:** 2013-03-26

**Authors:** Ursula Kirchmayer, Mirko Di Martino, Nera Agabiti, Lisa Bauleo, Danilo Fusco, Valeria Belleudi, Massimo Arcà, Luigi Pinnarelli, Carlo Alberto Perucci, Marina Davoli

**Affiliations:** 1 Department of Epidemiology, Lazio Regional Health ServiceRome, Italy; 2 National Agency for Regional Health ServicesRome, Italy

**Keywords:** infarction, prevention, drugs, mortality, epidemiology, pharmacoepidemiology

## Abstract

**Purpose:**

There are some methodological concerns regarding results from observational studies about the effectiveness of evidence-based (EB) drug therapy in secondary prevention after myocardial infarction. The present study used a nested case–control approach to address these major methodological limitations.

**Methods:**

A cohort of 6880 patients discharged from hospital after acute myocardial infarction (AMI) in 2006–2007 was enrolled and followed-up throughout 2009. Exposure was defined as adherence to each drug in terms of the proportion of days covered (cutoff ≥ 75%). Composite treatment groups, that is, groups with no EB therapy or therapy with one, two, three, or four EB drugs), were analyzed. Outcomes were overall mortality and reinfarction. Nested case–control studies were performed for both outcomes, matching four controls to every case (841 deaths, 778 reinfarctions) by gender, age, and individual follow-up. The association between exposure to EB drug therapy and outcomes was analyzed using conditional logistic regression, adjusting for revascularization procedures, comorbidities, duration of index admission, and use of the study drugs prior to admission.

**Results:**

Mortality and reinfarction risk decreased with the use of an increasing number of EB drugs. Combinations of two or more EB drugs were associated with a significant protective effect (*p* < 0.001) versus no EB drugs (mortality: 4 EB drugs: OR_adj_ = 0.35; 95%CI: 0.21–0.59; reinfarction: 4 EB drugs: OR_adj_ = 0.23; 95%CI: 0.15–0.37).

**Conclusions:**

These findings of the beneficial effects of EB polytherapy on mortality and morbidity in a population-based setting using a nested case–control approach strengthen existing evidence from observational studies. Copyright © 2013 John Wiley & Sons, Ltd.

## INTRODUCTION

Guidelines for drug treatment in clinical practice are based on evidence from clinical trials performed on selected populations.^1^,^2^ In recent years, the usefulness of observational studies for investigating the effectiveness of drugs in real-world settings has been widely recognized.^3^ Observational studies offer several advantages compared with clinical trials, particularly with respect to external validity: basing a study on a large population makes it more representative and allows for generalization. Thus, the results can be applied to medical practice in real-life settings because patients included in the cohort are much more similar to the resident population in terms of factors that may influence the efficacy of treatment, such as gender, age, comorbidities or polypharmacy. Finally, observational studies allow researchers to evaluate combined drug therapies that are recommended by guidelines and prescribed to patients in clinical practice but which are not addressed via clinical trials.

The availability of data from health information systems is an important milestone for population-based comparative effectiveness research in the field of pharmacoepidemiology.^4^ However, analyzing the associations between drug exposure and health outcomes carries the risk of specific methodological pitfalls that may lead to erroneous results, especially when both drug treatment and outcomes are measured in the same time window.^5^ Even though important progress has been achieved in recent years, for example, regarding immortal time bias,^6^ other critical aspects, such as bias due to changes in adherence over time, remain the subject of scientific discussion.

In the context of secondary prevention after acute myocardial infarction (AMI), several research groups have investigated the effects of evidence-based (EB) drug therapy on mortality and morbidity.^7^–^17^ However, each of these observational studies had some limitations with respect to internal validity, and the studies yielded conflicting results. Most researchers used drug therapy prescribed at discharge from hospital as a proxy measure for drug exposure without having any information about actual adherence during the follow-up period. Notably, this use of “intention-to-treat” information carries the risk of exposure misclassification.^7^,^10^,^12^–^15^ In other studies, the method for measuring adherence to therapy was not clearly explained,^9^ or drug intake was estimated through patients' self-reporting.^11^ Two US studies were based on outpatient prescription records. In one of them, drug intake was not considered exposure but was instead considered a covariate for adjusting the mortality temporal trend.^8^ The other study did not consider combined drug therapy, analyzing single drugs and their relation to mortality.^17^ Both studies used databases of selected patients, which limited external validity. The definition of exposure varied, and different combinations of single drugs were considered. The follow-up varied between 6 months and 12 years, with most studies focusing on periods of 1 year or less.^10^,^12^–^16^ Moreover, studies in which both drug treatment and outcomes were measured in the same time frame did not describe whether and how immortal time bias and bias due to changes in adherence over time were considered and taken into account.^9^,^11^,^16^,^17^

The present study used an innovative approach in an effort to overcome the major methodological limitations of previous observational studies. Specifically, a nested case–control study with incidence-density sampling was performed to estimate the association between adherence to EB drug therapy for secondary prevention after AMI and survival and incidence of a new AMI.

## METHODS

**Figure 1:**
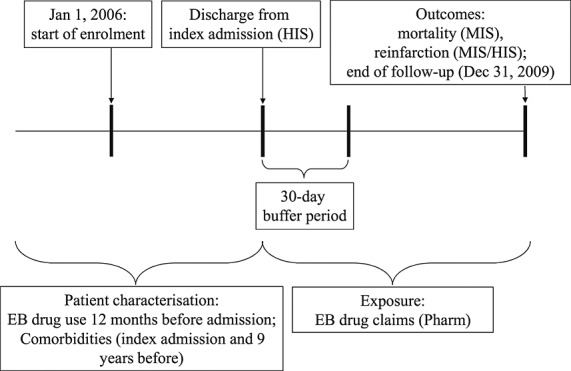
Study design: enrolment, follow-up, exposure, and outcomes. EB, evidence-based; HIS, Hospital Information System; MIS, Mortality Information System; Pharm, drug claims registry

The main elements of the study design are shown in Figure 1.

### Data sources

Our department has access to regional health information systems that contain mortality, hospital admission, and drug claims data. The details of the individual systems are reported in the Appendix.

### Setting and study population

The present observational study was based on the population living in the Lazio region of Italy, which comprises about five million persons. Using data from the regional Hospital Information System (HIS), the study enrolled a cohort consisting of all patients discharged from hospitals between 1 January 2006 and 30 November 2007, with a diagnosis of AMI (index admission). AMI was defined as a primary diagnosis of ICD-9-CM codes 410.xx or a primary diagnosis of an AMI-related condition along with a secondary diagnosis of 410.xx (Appendix).

Patients aged 35–100 years at discharge were considered for inclusion in the study. Only incident cases of AMI were included. Patients with hospital admission during the previous 9 years for infarction, percutaneous coronary intervention (PCI), bypass, coronary disease, or surgery of the heart and great vessels were excluded. Patients who were not registered in the regional health assistance file were excluded, as they could not be retrieved from the regional health information system (note that assistance is offered to all resident citizens without restriction). Finally, patients who spent more than half of their individual follow-up in the hospital and those with fewer than 30 days in an outpatient regimen were excluded, as they were considered extremely complex or instable patients. The patients are described in greater detail elsewhere.^18^

### Follow-up

Individual follow-up was considered to start on the first day after discharge from index admission. The end of the observation period was considered to be either the end of the study period (31 December 2009) or the date of an event, whichever occurred first. Consequently, the potential observation period varied between 2 and 4 years.

### Drug exposure

Exposure information was collected from the regional registry of all drugs dispensed by public and private pharmacies (Pharm); this registry is described in detail elsewhere and in the appendix.^18^ All drugs in this study were included in the patients' health care plans and are equally available to all residents in accordance with the universal health care coverage provided to residents of Italy.

Drug exposure was defined on the basis of recommendations by international and national guidelines for secondary prevention after AMI.^1^,^2^ Information about prescriptions of platelet aggregation inhibitors Anatomical Therapeutic Chemical (ATC) classification system: B01AC04, B01AC05, B01AC06), beta blocking agents (ATC: C07), agents acting on the renin-angiotensin system (ATC: C09), and HMG-CoA reductase inhibitors (ATC: C10AA) were retrieved for all patients.

Adherence was calculated according to the proportion of days covered (PDC) on the basis of the defined daily doses (DDDs) and was calculated separately for each drug. The choice to use this approach was based on preliminary research.^19^ Patients were defined as adherent when 75% or more of their individual follow-up was covered by a daily dose of the medication (i.e., PDC ≥ 75%). Inpatient regimens were excluded from this calculation because drugs are dispensed by the facility during inpatient treatment and thus cannot be retrieved from the Pharm database.

The following treatments were considered in the analysis: no EB drug therapy (<75% PDC of any of the drugs) and therapy with one, two, three, or four EB drugs. Sensitivity analyses were performed using a 50% cutoff for PDC and 50% and 75% cutoffs for the pill-count approach.

### Outcomes

Two outcomes were defined for the purpose of the analysis: mortality (all natural causes: ICD-9-CM < 800) identified through the regional Mortality Information System (MIS) database and reinfarction (either mortality, ICD-9-CM 410–414, or hospital admission for AMI, according to the inclusion criteria, whichever happened first). The first 30 days after discharge were considered a buffer period to give all patients the chance to achieve clinical stability and to guarantee a minimum observation period of 1 month.

### Study design and data analysis

Two nested case–control analyses were performed separately for mortality and reinfarction. Patients with study outcomes during follow-up were defined as cases. Four controls were selected for each case that were matched for age (5-year groups), gender, and time since AMI using incidence density sampling, thus ensuring an equal time window for measuring drug exposure for cases and controls.^20^,^21^

The association between adherence to EB drug therapy and outcomes was analyzed using a conditional logistic regression model. Potential confounders were selected in two steps. First, a list of potential risk factors were selected on the basis of *a priori* knowledge of the disease, including the following: duration of index admission, revascularization procedures during the index admission (PCI or bypass), 17 comorbidities retrieved from hospital records both for index admission and during the 9 previous years (Appendix), and use of the study drugs during the 12 months prior to index admission (defined as at least two prescriptions).

Second, the *a priori* risk factors were further selected through a bootstrap stepwise procedure, separately for mortality and reinfarction, to determine which factors were actually associated with the outcomes of interest.^22^ With the use of this approach, 1000 replicated bootstrap samples were selected from the original cohort. A bootstrap sample is a sample of the same size as the original dataset chosen with replacement. Thus, a given subject in the original cohort may occur multiple times, only once, or not at all in a specific bootstrap sample. A stepwise procedure with thresholds of *p* = 0.05 for variable selection and for variable elimination was applied to each replicated sample, and only the risk factors selected in at least 50% of the procedures were included as confounders in the conditional logistic regression models. The factors included in the two final models are reported in footnotes to the tables.

Odds ratios (ORs) and 95% confidence intervals (95%CIs) were calculated with “no EB therapy” defined as the reference group to which all other categories were compared. In sensitivity analysis, the following reference groups were also tested: no EB therapy + 1 EB drug vs. 2, 3, and 4 EB drugs; ≤2 EB drugs vs. 3–4 EB drugs; no EB therapy vs. 1–2 EB drugs, and 3–4 EB drugs. Differences between individual groups were investigated (3 vs. 2 EB drugs, 4 vs. 3 EB drugs). Finally, the potential effect of modification by time since AMI was investigated. The observation period was divided into tertiles separately for the two nested case–control analyses, and an exposure by tertile interaction was included in the conditional logistic regression models.

## RESULTS

**Figure 2:**
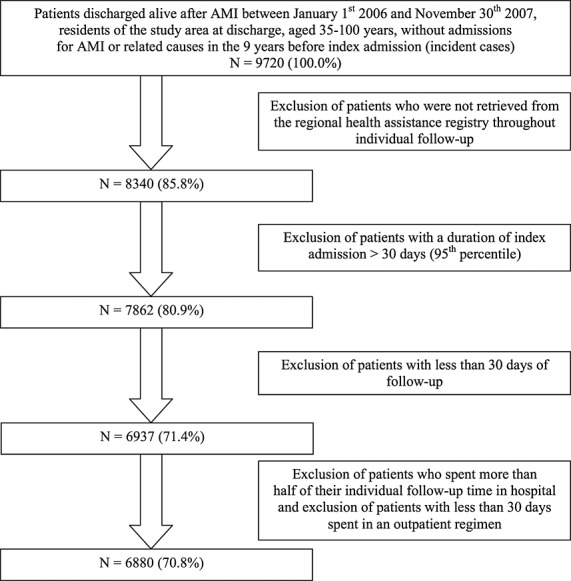
Cohort selection. AMI, acute myocardial infarction

Of the initial 9720 resident patients discharged alive after a first AMI in the enrolment period who were aged 35–100 years at discharge, 6880 patients were enrolled in the study cohort (Figure 2). Of these, 67.5% were men. The mean age was 72.5 years for women and 63.7 years for men (Table 1). The median follow-up was 994.5 days. Between 60% and 70% of the patients used antiplatelets, angiotensin-converting-enzyme (ACE) inhibitor inhibitors/sartans, or statins, whereas only 10% used beta blockers. Women were less likely to use the study drugs, confirming previous findings.^18^ Gender differences were observed regarding the incidence of the study outcomes, with higher rates among women for both mortality and reinfarction.

**Table 1:** Characteristics of the study cohort: age groups, exposure to drugs (patients with PDC ≥ 75%), mortality and reinfarction IR*

	Men		Women		Total	
			
	4642 (67.5%)		2238 (32.5%)		6880 (100.0%)	
Age groups (years) †	*N*	%	*N*	%	*N*	%
35–54	1155	24.9	204	9.1	1359	19.8
55–64	1288	27.7	345	15.4	1633	23.7
65–74	1226	26.4	579	25.9	1805	26.2
75–84	777	16.7	770	34.4	1547	22.5
85–99	196	4.2	340	15.2	536	7.8
Exposure to single drugs	*N*	%	*N*	%	*N*	%
Antiplatelet †	3375	72.7	1389	62.1	4764	69.2
Beta blockers ‡	491	10.6	205	9.2	696	10.1
ACE inhibitors/sartans ‡	2918	62.9	1371	61.3	4289	62.3
Statins †	3209	69.1	1224	54.7	4433	64.4
Outcomes	*N*	*IR* *	*N*	*IR* *	*N*	*IR* *
Mortality †	478	41.0	363	65.7	841	49.0
Reinfarction †	462	39.7	316	57.2	778	45.3

IR, incidence ratio; PDC, proportion of days covered.

* Rates for 1000 person-years.

† Difference between males and females statistically significant (*p* < 0.001).

‡ Difference between males and females not statistically significant (*p* > 0.05).

The nested case–control study for mortality was based on 841 cases, while the reinfarction study was based on 778 cases, half of which were fatal. The characteristics of the cases and controls of the two nested studies are reported in Table 2. For both substudies, the use of EB polytherapy was higher among controls, and only a very small group of patients used complete EB therapy. Controls had more frequently undergone PCI or bypass during index admission. The prevalence of comorbidities was higher among cases for almost all conditions, and the observed differences between cases and controls were similar in the two substudies. For both outcomes, cases had made use of study drugs before the event more often than controls, indicating a higher prevalence of pre-existing cardiovascular conditions. The results of the regression models are summarized in Table 3. With respect to the reference category (no EB therapy), crude ORs decreased with increasing number of drugs to 0.23 for mortality and for reinfarction. After adjusting for potential confounders, the results remained stable and the risk was significantly lower (*p* < 0.001) for both outcomes in patients using at least two of the recommended EB drugs. Adherence to complete EB polytherapy was associated with a risk reduction of dying of 65% (OR_adj_ 0.35, 95%CI 0.21–0.59, *p* < 0.001) and a risk reduction of reinfarction of almost 80% (OR_adj_ 0.23, 95%CI 0.15–0.37, *p* < 0.001).

**Table 2:** Characteristics of the two nested case–control populations

	Mortality				Reinfarction			
		
	Cases		Controls		Cases		Controls	
				
	*N*	%	*N*	%	*N*	%	*N*	%
	841		3329		778		3083	
Fatal					387		49.7	
Nonfatal					391		50.3	
Exposure								
No EB therapy	221	26.3	501	15.0	191	24.6	426	13.8
1 EB drug	199	23.7	677	20.3	173	22.2	536	17.4
2 EB drugs	224	26.6	999	30.0	209	26.9	926	30.0
3 EB drugs	176	20.9	968	29.1	176	22.6	951	30.8
Complete EB therapy	21	2.5	184	5.5	29	3.7	245	7.9
Interventions during index admission								
PCI	236	28.1	1429	42.9	284	36.5	1482	48.0
Bypass	10	1.2	76	2.3	6	0.8	75	2.4
Comorbidities (index admissions and 9 years before)								
Malignant neoplasm	210	25.0	428	12.9	96	12.3	309	10.0
Diabetes	262	31.2	546	16.4	248	31.9	488	15.8
Disorders of lipid metabolism/obesity	85	10.1	377	11.3	141	18.1	362	11.7
Hematologic diseases	171	20.3	327	9.8	113	14.5	265	8.6
Hypertension	445	52.9	1349	40.5	404	51.9	1119	36.3
Conduction disorders	123	14.6	380	11.4	92	11.8	338	11.0
Cardiac dysrhythmias	334	39.7	931	28.0	263	33.8	749	24.3
Heart failure	391	46.5	786	23.6	279	35.9	590	19.1
Other cardiac diseases	257	30.6	663	19.9	199	25.6	501	16.3
Cerebrovascular disease	262	31.2	663	19.9	186	23.9	522	16.9
Diseases of arteries,								
Arterioles and capillaries	448	53.3	1083	32.5	389	50.0	830	26.9
Chronic obstructive								
Pulmonary disease	176	20.9	463	13.9	146	18.8	334	10.8
Chronic nephropathies	214	25.4	384	11.5	180	23.1	308	10.0
Chronic liver, pancreas,								
Digestive diseases	54	6.4	124	3.7	46	5.9	126	4.1
Gastro-oesophageal								
haemorrhage	39	4.6	77	2.3	28	3.6	53	1.7
EB drug use 12 months before admission (2+ prescriptions)								
Antiplatelet	326	38.8	1048	31.5	269	34.6	802	26.0
Beta blockers	106	12.6	410	12.3	97	12.5	334	10.8
ACE-inhibitors/Sartans	464	55.2	1727	51.9	374	48.1	1412	45.8
Statins	120	14.3	382	11.5	126	16.2	350	11.4

EB, evidence based.

**Table 3:** Results of the logistic regression model for mortality and reinfarction: crude and adjusted ORs, 95%CIs, and *p*-values

EB drug therapy	OR crude	95%CI	OR adjusted *	95%CI	*p*-value
Mortality					
No EB therapy	1.00		1.00		
1 EB drug	0.66	0.53–0.83	0.68	0.53–0.87	0.003
2 EB drugs	0.49	0.39–0.61	0.59	0.47–0.76	<0.001
3 EB drugs	0.39	0.31–0.49	0.59	0.46–0.76	<0.001
Complete EB therapy	0.23	0.14–0.37	0.35	0.21–0.59	<0.001
Reinfarction					
No EB therapy	1.00		1.00		
1 EB drug	0.72	0.57–0.92	0.73	0.57–0.97	0.018
2 EB drugs	0.49	0.39–0.61	0.49	0.38–0.62	<0.001
3 EB drugs	0.38	0.30–0.48	0.37	0.28–0.47	<0.001
Complete EB therapy	0.23	0.15–0.35	0.23	0.15–0.37	<0.001

EB, evidence based; OR, odds ratio; CI, confidence interval.

* Potential confounders included in mortality analysis: PCI and bypass at index admission, heart failure, malignant neoplasm, disorders of lipoid metabolism/obesity, diabetes, chronic nephropathies, cerebrovascular disease, diseases of arteries, arterioles and capillaries, hemorrhagic stroke, hematologic diseases, cardiac dysrhythmias, duration of index admission.

Potential confounders included in re-infarction analysis: PCI and bypass at index admission, heart failure, diabetes, chronic nephropathies, diseases of arteries, arterioles and capillaries, ACE inhibitors/sartans before admission, duration of index admission.

The composite exposure indicators (one–three EB drugs) were investigated regarding the role of the single drugs. Low adherence to beta blockers accounted for the failure to reach sufficient PDC at all levels. This was particularly evident in the group that used three EB drugs: in this group, about 90% of patients failed to be defined as patients treated with complete EB therapy because of missing doses of beta blockers (results not shown).

The sensitivity analyses considered PDC at a lower cutoff (50%) as well as pill count, assuming a dosage of one pill per day for each single drug and using both the 50% and the 75% cutoffs. In all cases, the risk reduction associated with adherence to EB therapy was slightly lower, but the general results remained stable: the ORs for mortality using complete EB polytherapy were as follows: PDC 50%: OR_adj_ 0.41, 95%CI 0.28–0.61, *p* < 0.001; pill count 75%: OR_adj_ 0.53, 95%CI 0.38–0.73, *p* < 0.001; pill count 50%: OR_adj_ 0.51, 95%CI 0.37–0.70, *p* < 0.001. The results for reinfarction were similar.

Sensitivity analysis using different reference and comparison groups showed risk reductions for the use of an increasing number of drugs that were similar to those summarized in Table 3. Testing for differences between single groups showed the significantly lower mortality of patients taking four EB drugs compared with patients taking three EB drugs (*p* = 0.043) as well as significant differences in reinfarction for use of 3 vs. 2 EB drugs (*p* = 0.015) and for use of 4 vs. 3 EB drugs (*p* = 0.052) (detailed results not shown).

Finally, accounting for potential effect modification by time since AMI, the protective effect of polytherapy was confirmed in the short-term (observation time less than the first tertile), medium-term (observation time between the first and the second tertiles), and long-term (observation time greater than the second tertile) observations for both outcomes. The interaction terms were not statistically significant (*p* = 0.878 for mortality and *p* = 0.951 for reinfarction).

## DISCUSSION

The present nested case–control study provides clear evidence that EB drug therapy is associated with reductions in mortality and reinfarction after first AMI in a population-based setting. For patients treated with a combination of four EB drugs as recommended by guidelines, long-term mortality was associated with a risk reduction of 65%, and reinfarction was associated with a risk reduction of almost 80%.

Our results confirm findings from clinical trials on single or multiple drugs. A summary of the scientific evidence on mortality and reinfarction, as reported in the context of WHO's MONICA program, consistently favors the use of beta blockers, antiplatelet drugs, and ACE inhibitors.^23^ Moreover, there is solid evidence from clinical trials regarding the benefits of adding statins to the drug regimen.^24^ Direct comparison with the reductions observed in clinical trials cannot be made as no trials have investigated the combined therapy that we investigated in this study.

Previous observational studies yielded conflicting results, with some authors reporting no significant differences in mortality for use of EB drug therapy compared with the use of no drug therapy and others reporting beneficial effects. Of the studies of EB combination therapy, the majority reported reductions in mortality of 46% to 97% among users of optimal therapy,^10^–^16^ whereas other studies did not detect significant differences.^7^,^9^

Comparisons between studies is not straightforward because of the considerable methodological differences in setting (routine data vs. survey), cohort composition (hospital records vs. registries), exposure definition (multiple drugs vs. single agents), measurement (as-treated analysis vs. intention-to-treat analysis), outcome definition (administrative data vs. self-reporting), length of follow-up (from 6 months to 12 years), and confounding controls (clinical data vs. age and gender only).

The present study was conducted to try to overcome some of the major limitations of previous observational studies and to produce valid long-term population-based results. The exposure measure was chosen on the basis of previous methodological considerations.^19^ The applied measure was quite conservative, defining adherence to complete EB therapy on the basis of PDC ≥ 75% for each drug to minimize false positives for adherence. In fact, the prevalence of use of EB polytherapy that was estimated in the present study was considerably lower and is not directly comparable with the results on adherence obtained in our previous study using a different exposure measure and 12-month follow-up.^18^

Calculating therapeutic coverage through the DDD carries the risk that we are not accounting for real-life dosing of a drug when it is used for other than its principal indication. This is the case for beta blockers in the present study, which is evident in Table 1 and was confirmed by a detailed analysis of the composition of the groups of patients using two, three, or four EB drugs (results not shown). Evidently, in our study, beta blockers were prescribed at doses lower than the DDD for secondary prevention after AMI. Unfortunately, information on daily doses prescribed to individual patients was not available.

A major challenge in this type of observational study is that both exposure and outcomes are measured in the same period. This implies that the onset of an outcome and its timing affect the drug regimen classification. When the exposure time overlaps the follow-up time, patients who die or experience outcomes early during the exposure measurement period are less likely to obtain the study drugs and, as such, are more likely to be classified as nonexposed. This leads to overestimation of any beneficial treatment effect.^25^ On the other hand, patients who have already begun therapy and experience early outcomes during the exposure measurement period are more likely to be classified as adherent to treatment. In fact, the probability of complying with drug therapy after AMI decreases over time.

Analysis of the original cohort showed that the proportion of patients who was adherent to EB polytherapy varied from 44% in the first 6 months after infarction to 33% in the fourth 6-month period (results not shown). Similar observations were reported in an Italian primary care study on adherence to pharmacological therapy after AMI, which showed that a significant number of patients discontinued treatment over time.^26^ When using traditional techniques based on the standard survival analysis, the reduction in compliance over time leads to underestimation of any beneficial adherence effect. This systematic error can be termed “change in adherence bias.” This kind of bias was counterbalanced in the present nested case–control analysis because we used a risk-set control sampling that attributes the same length of observation to cases and to their matched controls to ensure equal time windows to measure exposure.^27^ Matching for age and gender led to very small differences between the unadjusted and the adjusted results. This confirms previous findings that age and gender account for a substantial part of adjustment.^28^

Finally, sensitivity analyses confirmed the robustness of the present results. With the use of exposure categories as defined in a previous study,^9^ our results remained stable. The risk reduction was similar, but in our study, the adjusted results were statistically significant, whereas in the Austrian study, adjustment for age and gender abrogated the benefit related to multiple-drug combination therapy.^9^

The major limitation of the present study is that it is impossible to control for some potential confounders, especially factors determined by lifestyle (e.g., smoking) or clinical information (e.g., body mass index and severity of AMI). The data in Table 2 show that cases who died or experienced reinfarction had fewer PCIs or coronary bypass interventions during their in-hospital stay and were affected by more comorbidities. We accounted for these differences by adjusting for all available potential confounders, but it is likely that the lack of more detailed clinical data might have caused residual confounding. We tried to counteract this limit by applying a number of restrictions to obtain a cohort with patients that were as homogeneous as possible. Notably, the robustness of our results in the sensitivity analyses, the evident trend of efficacy with increasing number of drugs, and the agreement with the results of clinical trials^1^,^2^,^23^,^24^ and other observational studies^10^–^16^ support our finding of an overall beneficial effect.

There are some things to keep in mind concerning the use of stepwise procedures for selecting potential confounders. The original list of potential confounders was defined on the basis of *a priori* knowledge about the disease and risk factors. Bootstrap stepwise is just a way to improve the efficiency of the statistical models used to control confounding. In fact, this procedure allows us to determine which of the *a priori* risk factors are actually associated with the outcome in the specific context of our data.^22^ This allows us to exclude those factors from the models that do not act as confounders because they are not associated with the outcome; this avoids overparameterization and improves estimator efficiency.

## CONCLUSION

The present study provides evidence of the medium-term and long-term beneficial effects of combined EB drug therapy as secondary prevention after AMI in a real-life setting. The study methodology overcomes most of the limitations of observational studies published on this topic thus far.

**KEY POINTS**

- Clinical guidelines on secondary prevention after acute myocardial infarction (AMI) with combined drug therapy are based on evidence from clinical trials.
- Results from observational studies on this topic are a matter for discussion because of methodological concerns.
- A new methodology was applied to overcome limitations of previously published observational studies.
- Our study results provide evidence for medium-term and long-term beneficial effects of combined drug therapy after AMI in secondary prevention in real-life settings.

## SUPPORTING INFORMATION

Appendix

Data sources
Algorithm for selection of the cohort
Selection of comorbidities from hospital discharge records
