# Supplementary material for: Effect of evidence-based drug therapy on long-term outcomes in patients discharged after myocardial infarction: a nested case–control study in Italy
Source: Pharmacoepidemiol Drug Saf. 2013 Mar 26;22(6):649–57. doi: 10.1002/pds.3430 (PMC3746119; doi:10.1002/pds.3430)
Supplement: Supplementary file 1 [file pds0022-0649-SD1.pdf]

Appendix

Data sources

Hospital Information System (HIS)

The HIS includes patient characteristics (patient fiscal code, gender, date and place of birth, place of residence); admission and discharge dates; discharge diagnoses (up to 6); procedure codes (up to 6) according to the International Classification of Disease, Ninth Revision, Clinical Modification (ICD-9-CM); ward(s) of stay; date(s) of in-hospital transfer; and a regional code that corresponds to the admitting facility.

Mortality Information System (MIS)

The MIS includes patient demographic characteristics (name, age, gender, fiscal code, place and date of birth, residence, marital status, occupation), as well as date, place, and cause of death (codified by ICD-9 codes).

Drug claims registry (Pharm)

Pharm comprises individual records for each medical prescription dispensed in public and private pharmacies within the territory of the local health authorities for the resident population. The registry is limited to those drugs prescribed for outpatient use that are reimbursed by the health care system. The drugs in the study are all covered in Pharm. In this registry, drugs are identified by the national drug registry code, which refers to the international ATC classification and allows for the exact quantification of the dispensed drug. Individual patient data (patient fiscal code) and the date the drug is dispensed are reported for every prescription.

For Review Only

Algorithm for selection of the cohort

ICD-9-CM codes for the identification of AMI cases:

Primary diagnosis of acute myocardial infarction (ICD-9-CM 410.xx) or secondary diagnosis of AMI associated with one of the following conditions as the primary diagnosis:

ICD-9-CM code	Condition
411	Other acute and subacute forms of ischemic heart disease
413	Angina pectoris
414	Other forms of chronic ischemic heart disease
423.0	Hemopericardium
426	Conduction disorders
427	Cardiac dysrhythmias, excluding 427.5 Cardiac arrest
428	Heart failure
429.5	Rupture of chordae tendineae
429.6	Rupture of papillary muscle
429.71	Acquired cardiac septal defect
429.79	Certain sequelae of myocardial infarction not elsewhere classified
429.81	Other disorders of papillary muscle
518.4	Acute edema of lung, unspecified
518.81	Acute respiratory failure
780.01	Coma
780.2	Syncope and collapse
785.51	Cardiogenic shock
799.1	Respiratory arrest

For Review Only

997.02	Iatrogenic cerebrovascular infarction or hemorrhage
998.2	Accidental puncture or laceration during a procedure

For Review Only

Selection of co-morbidities from hospital discharge records

Condition	ICD-9-CM codes	
	Index admission	Admissions during the 9 years prior to index admission
Malignant neoplasms	140.0–208.9	140.0–208.9
Diabetes		250.0-250.9
Disorders of lipoid metabolism/obesity		272, 278.0
Hematologic diseases	280-285, 288, 289	280-285, 288, 289
Hypertension		401-405
Heart failure	428	428
Other cardiac diseases	393-398, 423 (excl. 423.0), 424, 425, 745, 746.3-746.6, V15.1, V42.2, V43.2, V43.3, V45.0,	093.2, 391, 393-398, 420, 421, 422, 423, 424, 425, 429 (excl. 429.7), 745, 746.3-746.6, V15.1, V42.2, V43.2, V43.3, V45.0
Conduction disorders	426	426
Cardiac dysrhythmias	427, 785.0, 996.01, 996.04, V45.0, V53.3	427, 785.0, 996.01, 996.04, V45.0, V53.3
Cerebrovascular disease	430-438	430-438
Hemorrhagic stroke	430, 431, 432	430, 431, 432
Ischemic stroke/ transient cerebral ischemia	433.x1, 434, 435.x, 436	433.x1, 434, 435.x, 436
Diseases of arteries, arterioles, and capillaries	440-448 (excl 441.1, 441.3, 441.5, 441.6, 444)	440-448, 557
Chronic obstructive pulmonary disease (COPD)		491-492, 494, 496
Chronic nephropathies	582-583, 585-588, V42.0,	582-588, V42.0, V45.1,

For Review Only

	V45.1, V56<br>*procedures* 38.95, 39.95, 54.98	V56<br>*procedures* 38.95, 39.95, 54.98, 55.6
Chronic liver, pancreas, and digestive diseases	571-572, 577.1-577.9, 555, 556, V42.7	571-572, 577.1-577.9, 555, 556, 0.70, 456.0-456.2, 570, 573, V42.7
Gastro-esophageal hemorrhage	530.2 (2009: 530.21), 530.82, 531.0, 531.2, 531.4, 531.6, 532.0, 532.2, 533.4, 533.6, 534.0, 534.2, 534.4, 534.6, 535.01, 535.11, 535.21, 535.31, 535.41, 535.51, 578.9	530.2 (2009:530.21), 530.82, 531.0, 531.2, 531.4, 531.6, 532.0, 532.2, 533.4, 533.6, 534.0, 534.2, 534.4, 534.6, 535.01, 535.11, 535.21, 535.31, 535.41, 535.51, 578.9

For Review Only
